# Activity of clinically relevant antimalarial drugs on *Plasmodium falciparum* mature gametocytes in an ATP bioluminescence “transmission blocking” assay

**DOI:** 10.1186/1475-2875-11-S1-P7

**Published:** 2012-10-15

**Authors:** Joël Lelièvre, Maria Jesus Almela, Sonia Lozano, Celia Miguel, Virginia Franco, Didier Leroy, Esperanza Herreros

**Affiliations:** 1 GiaxoSmithKiine R&D, Tres Cantos Medicine Development Campus, Malaria Discovery Performance Unit, C/Severo Ochoa, 2, 28760 Tres Cantos, Madrid, Spain; 2 Present address: Centro de Bioiogia Molecular (UAM-CSIC), C/Nicoiás Cabrera, 1, Campus de la Universidad Autónoma de Madrid, 28049 Madrid, Spain; 3 Medicines for Malaria Venture. 20, route de Pré-Bois. 1215 Geneva 15, Switzerland

## Background

Current anti-malarial drugs have been selected on the basis of their activity against the symptom-causing asexual blood stage of the parasite. How many of these drugs also target gametocytes, the sexual stage responsible for disease transmission, remains unknown. Blocking transmission is one of the main strategies in the eradication agenda and implies the identification of new molecules active against gametocytes. However, so far, the main limitation for measuring the effect of molecules against mature gametocytes on a large scale is the lack of a standardized and reliable method. Here we provide an efficient method to produce and purify mature gametocytes *in vitro.* Based on this new procedure, we developed a robust, affordable and sensitive ATP bioluminescence-based assay. We then assessed the activity of 17 gold-standard anti-malarial drugs on *Plasmodium* late stage gametocytes.

## Materials and methods

Difficulties in producing large amounts of gametocytes have limited progress in the development of malaria transmission blocking assays. We improved the method established by Ifediba and Vanderberg to obtain viable, mature gametocytes en masse. We designed an assay to determine the activity of antimalarial drugs based on the intracellular ATP content of purified stage IV-V gametocytes after 48h of drug exposure in 96/384-well microplates. Measurement of drug activity on asexual stages and cytotoxicity on HepG2 cells were also obtained to estimate the specificity of the active drugs. The methodology is fully described at Lelièvre et al[1]. 

## Results

The assay was validated by comparing traditional microscopy examination with the ATP bioluminescence assay using a set of 6 anti-plasmodial drugs. We obtained comparable IC_50_ values with both methods.

After validation, 16 clinically relevant antimalarial drugs presenting different mechanism of action were tested. Only epoxomicin (0.42 nM) and methylen blue (0.49 µM) showed IC_50_ values in the range of nanomolar.

Epoxomicin is an inhibitor of proteasome activity, exerting a toxic effect on the parasite, but as this function is also essential in mammalian cells its cytotoxicity is of concern.

Primaquine has been reported to destroy the inner structure of *P.falciparum* mitochondria. It is assumed that primaquine activity depends on the formation of metabolites, more active than the parent compound. This 8-aminoquinoline has long been known to reduce the prevalence of circulating gametocytes in the peripheral bloodstream of patients. Due to the absence of the active metabolites involved in its mechanism of action, primaquine remains inactive *in vitro* (20.9 µM). Methylene blue (MB) was identified as a specific inhibitor of *P. falciparum* glutathione reductase, blocking heme polymerization within the food vacuole. Our study evaluated for the first time the IC_50_ of MB on stage IV-V gametocytes and HepG2 cell line (6.52 µM), founding a good activity but quite high cytotoxicity.

## Conclusions

The work described represents another significant step [2] towards determination of activity of new molecules on mature gametocytes with an automated assay suitable for medium/high-throughput screening. Considering that the biology of the sexual stages is very different from asexual forms, screening of compound libraries would allow us to discover novel anti-malarial drugs to target gametocyte-specific metabolic pathways.

## References

[B1] Lelievre J Almela MJ Lozano S Miguel C Franco V Leroy D Herreros E Activity of clinically relevant antimalarial drugs on *Plasmodium falciparum* mature gametocytes in an ATP bioluminescence “transmission blocking” assay PLoS One 7 e35019 22514702 10.1371/journal.pone.0035019 PMC3325938

[B2] Adjalley SH Johnston GL Li T Eastman RT Ekland EH Eappen AG Richman A Sim BK Lee MC Hoffman SL Fidock DA Quantitative assessment of *Plasmodium falciparum* sexual development reveals potent transmission-blocking activity by methylene blue Proc Natl Acad Sci U S A 2011 108 E1214 1223 10.1073/pnas.1112037108 22042867 PMC3223476

